# Fibroblast Growth Factor-1 vs. Fibroblast Growth Factor-2 in Ischemic Skin Flap Survival in a Rat Animal Model

**Published:** 2016-09

**Authors:** Ehsan Fayazzadeh, Hana Yavarifar, Seyyed Reza Rafie, Sadrollah Motamed, Maryam Sotoudeh Anvari, Mohammad Ali Boroumand

**Affiliations:** 1 Tehran Heart Center, Tehran University of Medical Sciences, Tehran, Iran;; 2 School of Medicine, Ankara University, Ankara, Turkey;; 3 Department of Plastic and Reconstructive Surgery, 15 Khordad Hospital, Shahid Beheshti University of Medical Sciences, Tehran, Iran

**Keywords:** Acidic fibroblast growth factor, Basic fibroblast growth factor, Ischemic necrosis, Skin flap survival, Rat

## Abstract

**BACKGROUND:**

One of the main challenges in skin flap surgery is tissue ischemia and following necrosis. The present study compares the effects of fibroblast growth factors 1 and 2 on increasing cutaneous vasculature, improving ischemia, and preventing distal necrosis in ischemic skin flaps in rat model.

**METHODS:**

Thirty rats were allocated into 3 groups (n=10) and 2×8 cm dorsal random-pattern skin flaps were raised after four daily subdermal injections of normal saline (control group), fibroblast growth factor 1 (FGF-1 group; 2.5 µg/day), or fibroblast growth factor 2 (FGF-2 group; 2.5 µg/day) at designated flap areas. Skin flap viability and number of blood vessels were evaluated on day 10 after elevation by planimetric analysis and histological examination.

**RESULTS:**

It was shown that administrations of FGF-1 and FGF-2 significantly decreased the percentage of flap necrosis and improved the percentage of ischemic survivable area, compared to the control samples. Meanwhile, the differences between these factors in terms of preventing skin flap necrosis and improving ischemia were also significant. The number of visible blood vessel sections was also higher in FGF-1 and FGF-2 groups than in the control group.

**CONCLUSION:**

These findings suggest that, while FGF-2 is still much more potent than FGF-1, treatment with either of these drugs could be very effective in increasing the survival of surgical flaps at risk (length to width ratio>3) in situations that other therapeutic options could not be considered.

## INTRODUCTION

One of the main challenges in skin flap surgery is tissue ischemia and necrosis. Compromised tissue perfusion may result in delaying or impairment of healing process, particularly in various wound cases such as diabetic ulcer, bed sore, skin flaps etc.^1^ Acceleration and/or enhancement of angiogenesis is a major therapeutic goal in many of recent therapeutic investigations to decrease necrosis in ischemic tissues.^2^ Studies of angiogenic materials including various subclasses of fibroblast growth factors (FGF) have revealed the potency of these factors in improving tissue survival in ischemic skin flaps by new blood vessel formation.^2^^-^^7^ However, many of these studies had equivocal findings, based on dosing of the substances used or timing of their administration. Therefore, no general agreement exists on the optimal dosage, frequency, and duration of therapy; moreover, only a very few studies have directly compared the effects of angiogenic factors in preventing ischemic necrosis in skin flaps or elsewhere. The current experiment pursues the authors’ previous studies on a variety of angiogenic factors and substances accelerating the wound healing process and compares the potency of acidic fibroblast growth factor (aFGF) and basic fibroblast growth factor (bFGF), also known as FGF-1 and FGF-2 respectively, in improvement of ischemic skin flap survival.

## MATERIALS AND METHODS

All steps of the current study were performed in accordance with the “Guide for the Care and Use of Laboratory Animals” (NIH publication No. 85-23, revised 1996). After institutional board review and ethical committee approval, 30 Sprague-Dawley male albino rats (weighing 250-300 g) were included in the experiment. Animals were housed in separate clean cages at a constant temperature of 20-24°C under 12h light/12h dark cycles and with free access to chow pellets and tap water. The specimens were under daily observance until the end of the study.

**Fig. 1:**
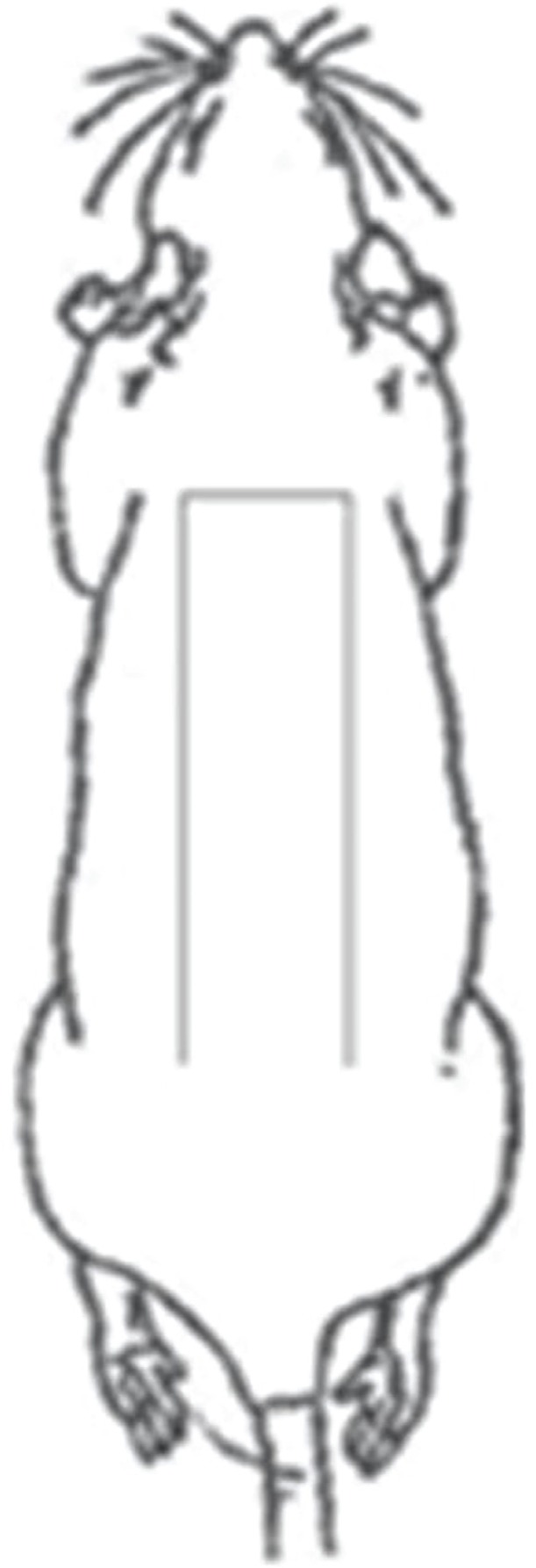
Outlines of skin incisions on dorsum of rat specimens, defining designated 4:1 flaps created 3 days later. Subdermal injections of substances were made at distal halves of designated flap areas

The flap creation was performed according to the model already described in a former article.^6^ The rats were anesthetized by intraperitoneal injections of ketamine hydrochloride (60 mg/kg) and xylazine (9 mg/kg) combination. After shaving skin and disinfecting the surgery area, full-thickness rectangular skin incisions (two 8 cm parasagittal incisions and one 2 cm cephalad horizontal incision) were made on dorsum of each animal (Figure 1) and then immediately sewed back in place without flap elevation. This was counted as day 0 of the intervention. 

Study specimens were then randomly distributed into three groups, with 10 samples in each group, as followed: group 1 (control), administration of 0.9% NaCl solution as group 2 (FGF-1), administration of 2.5 µg/day of acidic fibroblast growth factor as group 3 (FGF-2), and administration of 2.5 µg/day of basic fibroblast growth factor (both drugs from Peprotech, USA). All substances in study groups were injected subcutaneously (1 ml) in distal halves of designated flap areas, once per day, for 3 consecutive days after the initial operation. 

On day 3, the animals were reoperated and 4:1 caudally-based random-pattern skin flaps were created by undermining subdermal tissues in designated flap regions. The flaps included the entire thickness of skin and skin muscle (panniculus carnosus). Flap dimensions were 2×8 cm with their bases level with the location of iliac crests. During all operations, animals were kept on warming blankets and strict aseptic measures were observed.

On day 10, the animals were euthanized by lethal chloroform inhalation. Skin flaps were photographed and then excised. Flap necrosis was judged by black discoloration and induration of skin at cephalad ends. The margin between necrotic and cyanotic areas was determined using a magnifying loop. The areas of tissue necrosis (S_N_) and ischemia (S_I_) were calculated by Image J software (ver. 1.34, NIH, USA) and reported as relative percentages to the total flap area. 

Two skin samples (0.5×1.0 cm strips) including the wound margins were taken from each specimen at 2 cm and 4 cm away from the distal end of the flap area. Samples were fixed in 10% formalin, embedded in paraffin, and cut in 5 µm sagittal sections for hematoxylin and eosin staining. All samples were examined under high power (400X) light microscopy by two expert pathologists blinded to the study. The number of blood vessel sections was measured under 100X magnification. Fibroblast and endothelial cell proliferations were also evaluated by quantitative scoring. 

Data analysis was done by ANOVA one-way test using SPSS software (ver. 15.0 statistical software, SPSS Inc., Chicago, IL, USA). Bonferroni post hoc test was performed for multiple comparisons when applicable. Data are reported as mean±SD with *P* values less than 0.05 considered statistically significant. 

## RESULTS

**Fig. 2:**
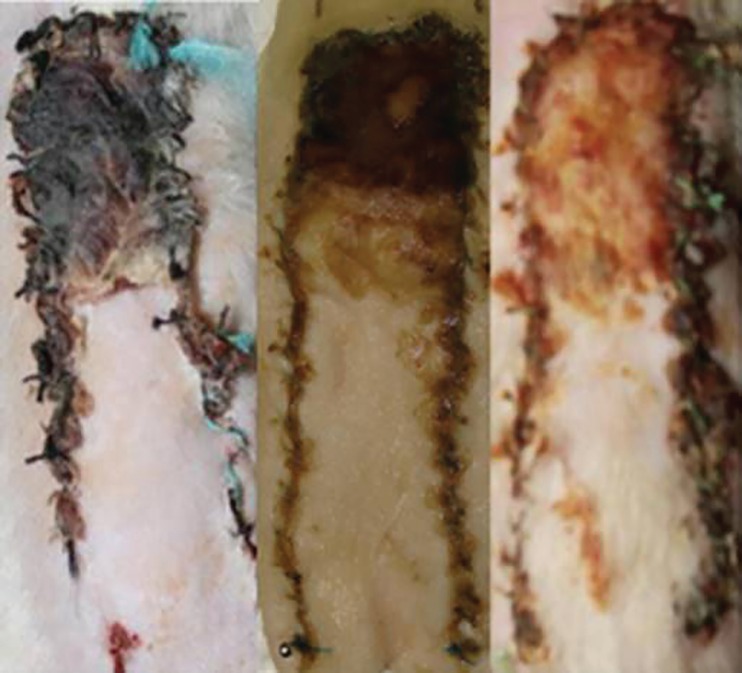
Necrotic (black) and cyanotic (reddish brown) discoloration in control (left), FGF-1 (center), and FGF-2 (right) specimens

Following flap creation on day 3, cyanotic/necrotic discoloration was evident at distal ends of the flaps in all study animals (Figure 2). Flap discolored area included a zone of apparent clinical necrosis (S_N_), as well as a zone of ischemic but still viable tissue (S_I_), as defined by skin color and stiffness besides light microscopy. On planimetric examination on day 10, the total area of ischemia plus necrosis (S_N_+S_I_) was nearly the same among study groups without any meaningful differences between them (45.61±7.47% in control group, 49.70±14.45% in FGF-1 group, 40.03±5.21% in FGF-2 group; *p*=0.108). However, necrosis in control group was statistically much more significant, compared to the other two groups (41.07±7.01% in control group, 24.16±9.95% in FGF-1 group, 7.67±1.79% in FGF-2 group; *p*<0.001). The difference between FGF-1 and FGF-2 groups was also statistically meaningful (*p*<0.001). 

Moreover, ischemic but viable zone in control group was significantly smaller than that in FGF-1 and FGF-2 groups (4.54±3.08% in control group, 25.55±7.10% in FGF-1 group, 32.36±4.34% in FGF-2 group; *p*<0.001). The difference between FGF-1 and FGF-2 groups also bore statistical significance (*p*<0.05). The percentage of the necrotic area relative to the total discoloration area was 90.20±6.29% in control group, 47.00±12.08% in FGF-1 group, and 19.13±3.43% in FGF-2 group (*p*<0.001, for comparison among 3 groups). 

Microscopic evaluation revealed injured epidermis in control group specimens. Furthermore, widespread aggregation of inflammatory cells and formation of granulation tissue was also evident. In FGF-1 and FGF-2 study groups, the degree of inflammatory cell infiltration and granulation tissue were exclusively lower, with variable extents of epidermal healing. Especially in FGF-2 group, fibrous tissue was well organized, occupying most of the thickness of dermis. The count of blood vessel sections under 400X magnification was also slightly higher in the latter two groups (48.26±4.83 in FGF-1 group and 51.75±5.47 in FGF-2 group), compared to the number of vessel sections in the control group (43.50±15.22); however, the differences between study groups did not reach statistical significance (*p*=0.093). 

## DISCUSSION

Improvement of wound healing and prevention of ischemic flap necrosis have been the goals of numerous studies, applying a wide variety of angiogenic substances and growth factors, since the introduction of skin flap animal model by McFarlane in 1965.^8^ Among these substances, prototypes of the fibroblast growth factor (FGF) family are proven to be implicated in tissue repair and wound healing^9^^-^^13^ and their applications in ischemic flaps survival have been accompanied by promising results.^3^^-^^5^^,^^14^^,^^15^

FGFs were amongst the earliest angiogenic factors discovered. They were found to stimulate endothelial cell proliferation, migration and differentiation in vitro and in vivo. However, the most important FGF prototypes in angiogenesis and wound healing including FGF-1 and FGF-2 lack a classical signal sequence that allows efficient export from cells. Thus, the role of endogenous FGFs in angiogenesis processes still remains uncertain. Some of these unresolved issues were recently taken up by several investigators, thus FGFs becoming once again the focus of angiogenesis research.^16^^,^^17^

The salvage of ischemic damage by administration of FGF-2 and FGF-1 in different animal models has been ascribed to increases in the vascularization of the ischemic zones by increasing the number of arterioles and capillaries.^15^^-^^18^ FGF-2 is generally regarded as one of the most potent angiogenic factors amongst the 23 members of the FGF family as it enhances proliferation and migration of macrophages, fibroblasts and endothelial cells towards injured tissues, induces collagen synthesis, promotes wound contraction, and increases formation of new epidermis, besides contribution in synthesis of extracellular matrix (ECM) constituents such as fibronectin and proteoglycan, and thus plays an important role in wound healing process.^9^^,^^13^^,^^19^

It is also believed that certain prototypes of the FGF family, including FGF-2, induce release of NO which yields endothelial-dependent relaxation in vascular shunts and improves perfusion and decreases tissue ischemia.^20^ FGF-1 has 50% homology with FGF-2, and although it is 10 times less potent as an angiogenic stimulant, it can provoke endothelial cell proliferation and motility and contribute to wound angiogenesis.^10^^,^^21^ In many studies using animal models of ischemic skin flaps, drugs were injected following flap elevation. In our experiment, we used the substances before raising the flaps to allow enough time to exert their angiogenic effects. 

**Fig. 3:**
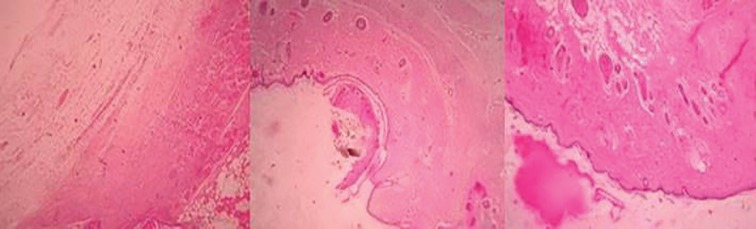
Photomicrographs of samples on day 10 show widespread granulation tissue, interspersed by fibroblasts in control group (left). In FGF-1 (center) and FGF-2 (right) groups, moderate-to-high degrees of fibroblast proliferation are evident in support of wound healing acceleration

The route of FGF administration also seems to be very important. In studies of myocardial ischemia, intravenous administration of FGF protein is less effective than intra-arterial injection (intracoronary), which suggests a stability or delivery problem of the protein. These results explain why, in some studies, FGF is less effective in improving ischemic revascularization. However, intrapericardial administration of FGF provides higher myocardial deposition and retention and lower systemic recirculation than intracoronary or intravenous routes.^18^ Hence, we preferred to inject the drugs in the distal half of the flap, an area supposed to be in maximal need for angiogenesis to take place. We designed a 4:1 flap length-to-width ratio in our study to generate significant necrosis in distal end of the flaps in control samples ubiquitously. As described in our previous article,^6^ two separable zones of discoloration could be distinguished in flap areas: a distal portion with black induration and other visual signs of necrosis; and a more proximal cyanotic segment with tissue ischemia but apparently still viable (Figure 3). 

Although the results of our studies did not show any significant variation in total area of discoloration including necrotic and ischemic zones among the groups, the reduction of necrotic zone in groups treated by FGF-1 and FGF-2 was considerable. Specifically in FGF-2 group specimens, the ischemic but still viable tissue comprised nearly all of the total discoloration area. In contrast, in the control group, the whole discolored area was consisted almost only of necrosis but no ischemia. In FGF-1 group, a well-defined outline was evident between the two zones of necrosis and ischemia. 

Although the reduction of necrotic tissue in FGF groups was impressive, the increase in vascular density in FGF-2 and FGF-1 groups did not lead to a significant improvement in the microscopic survey. This suggests that our criteria for analysis (visible vascular sections) may have been too gross to detect subtle differences. More accurate modalities such as laser Doppler and immunohistochemistry analysis would be hereby suggested for assessment of tissue perfusion and angiogenesis.

Studies using FGF-2 within the range of 2-2.7μg in single or multiple doses have all demonstrated favorable results in terms of improving flap survival.^6^^,^^15^ The data obtained from the current study support results from similar study.^6^ however, unlike the majority of experiments studying the effects of FGF-2 in ischemic flap survival, only a few have so far examined outcomes of FGF-1 application in animal models of skin flaps.^22^ Interestingly, the results of present study and our previous work using erythropoietin (100 U/kg) in a similar manner show that the extents of skin flap survival following administration of FGF-1 and erythropoietin are comparable to each other.^6^

To conclude, FGF-2 and FGF-1 were both shown to hinder progression of ischemia towards necrosis in skin flaps. Although FGF-2 has proven advantages over FGF-1 in preventing necrosis in ischemic regions of skin flaps, the latter factor has also significant positive impacts on ischemic skin flap survival.
